# A MOBILE CLINIC STUDY ON BRAIN HEALTH IN OLDER ADULTS IN A RURAL, SOCIOECONOMICALLY DISADVANTAGED COUNTY

**DOI:** 10.1093/geroni/igae098.4101

**Published:** 2024-12-31

**Authors:** Marianne Chanti-Ketterl, Jennifer Jones-Locklear, Jada Brooks, Cierra Williams, Misty Bullard, Keenya Locklear, Scott Davis, Heather Whitson

**Affiliations:** Duke University, Durham, North Carolina, United States; UNIVERSITY OF NORTH CAROLINA AT PEMBROKE, University of North Carolina Pembroke, North Carolina, United States; UNIVERSITY OF NORTH CAROLINA, Chapel Hill, North Carolina, United States; Duke University, Durham, North Carolina, United States; UNIVERSITY OF NORTH CAROLINA AT PEMBROKE, University of North Carolina Pembroke, North Carolina, United States; UNIVERSITY OF NORTH CAROLINA AT PEMBROKE, University of North Carolina Pembroke, North Carolina, United States; Duke University, Durham, North Carolina, United States; Duke University, Durham, North Carolina, United States

## Abstract

**Objective:**

This collaborative pilot project assessed the feasibility of enrolling 50 older adults from underrepresented populations in Robeson County, North Carolina (NC), considered a racially diverse and socioeconomically disadvantaged area.

**Methods:**

The study protocol, adapted from the Alzheimer’s Gut Microbiome Project, was modified to fit local cultural contexts with input from community and local academic partners. The Lumbee Tribe of NC, headquartered in Robeson County, and its Tribal IRB endorsed the project and community-informed recruitment materials. Participants were recruited through the NC Registry for Brain Health, community-based referrals, and local meetings. A mobile clinic facilitated study visits. After consenting, participants underwent immediate fasting glucose and cholesterol testing via finger prick and biomarker collection through venipuncture. They completed a Montreal cognitive assessment and answered demographics, lifestyle, medical conditions, environmental exposures, and diet questionnaires. Instructions for home stool sample collection and shipment were provided. A nurse then reviewed participants’ physical measures, offering health counseling and resources. Participants received a printed summary of most study results except those from venipuncture, which were sent to the lab. Compensation with merchant cards was provided after shipment of stool samples.

**Results:**

Fifty-seven individuals expressed interest; nine declined participation, two were ineligible, and five are pending the visit. To date, we have 41 people enrolled, nearly all recruited through community outreach.

**Discussion:**

This study demonstrated the feasibility of enrolling older adults in research from a rural, underserved area. The strong community-academic engagement, including endorsement from the Lumbee Tribe and using a local mobile clinic, was key to the recruitment process.

